# KCNJ11 (p.Arg192Cys)–associated MODY13 with coexisting autoimmunity in a child, a case report

**DOI:** 10.1186/s12887-026-06672-2

**Published:** 2026-03-02

**Authors:** Tarah H. Fatani

**Affiliations:** Department of Pediatrics, Pediatric Endocrinology, King Abdulaziz University, Jeddah, 21589 Saudi Arabia

**Keywords:** MODY13, KCNJ11 mutation, Pediatric diabetes, Autoimmunity, Monogenic diabetes, Sulfonylurea therapy

## Abstract

**Background:**

Maturity-onset diabetes of the young type 13 (MODY13) is a rare autosomal dominant monogenic diabetes caused by mutations in the potassium inwardly rectifying channel subfamily J member 11 (KCNJ11) gene. It can mimic both type 1 and type 2 diabetes mellitus (T1DM and T2DM), making accurate diagnosis challenging.

**Case presentation:**

We describe the case of a five-year-old boy with overlapping features of T1DM and T2DM. He presented with hyperglycemia, obesity, and acanthosis nigricans. These symptoms improved after losing 11 kg owing to lifestyle modifications. His hemoglobin A1c (HbA1c) level decreased from 9.6% to 5.8%, and the continuous glucose monitoring system (CGMS) showed 91% time in range (TIR). Eighteen months later, he developed postprandial hyperglycemia and tested positive for T1DM antibodies, prompting the administration of insulin at 0.7 U/kg/day. His glucose levels continued to fluctuate. Whole-exome sequencing identified a likely pathogenic KCNJ11 variant (NM_000525.3, c.574 C > T, p.Arg192Cys), confirming the diagnosis of heterozygous autosomal dominant MODY13. He was then started on oral glibenclamide at 0.2 mg/kg/day, which lowered his HbA1c level to 7.5% and raised his CGMS TIR readings to 70%; however, insulin dependence persisted because of ongoing autoimmune β‑cell destruction.

**Conclusions:**

This case demonstrates the diagnostic challenge in pediatric diabetes when the features of T1DM, T2DM, and MODY overlap. Prompt genetic testing can lead to precise therapy. The simultaneous appearance of MODY13 and autoimmunity suggests possible interactions between the gene and the immune system.

## Background

Maturity-onset diabetes of the young (MODY) is a rare form of autosomal dominant monogenic diabetes that impairs β-cell function and typically becomes apparent before a person reaches 25 years of age. It accounts for 1–5% of all pediatric diabetes cases worldwide. The condition usually presents as non-ketotic hyperglycemia, accompanied by a family history of dominantly inherited diabetes in at least two generations, negative islet autoantibodies, variable insulin requirements, and a lack of metabolic syndrome hallmarks, such as acanthosis nigricans, obesity, hypertension, fatty liver, or dyslipidemia [1–6].

MODY13, among the rarest subtypes, represents < 1% of all reported cases [7]. Bonnefond et al. first described MODY13 in 2012 [8], and < 35 cases have been reported worldwide. It is caused by a mutation in the potassium inwardly rectifying channel subfamily J member 11 (KCNJ11) gene located on chromosome 11p15. A heterozygous loss-of-function mutation results in haploinsufficiency, resulting in 50% normal protein levels, non-immune pancreatic β-cell dysfunction, and absolute deficiency of insulin secretion [1, 7, 9].

Family members with the same mutation can show variable phenotypic expression, ranging from neonates born with hyperinsulinemic hypoglycemia, neonatal diabetes mellitus (NDM), and developmental delay, Epilepsy, and Neonatal Diabetes (DEND) syndrome, which is marked by developmental delay, epilepsy, and NDM, to late-onset diabetes [10–13].

Misclassification of MODY as type 1 or type 2 (T1DM or T2DM) may result in unnecessary lifelong insulin therapy [3, 7, 14]. Although advances in genetic testing tools have improved recognition, most studies have focused on more common MODY subtypes, limiting MODY13 clinical spectrum exploration [4, 15, 16].

The ISPAD 2022 Guidelines emphasize the importance of genetic testing in atypical pediatric diabetes, especially in high‑consanguinity regions such as Saudi Arabia, to ensure precision therapy and spare patients from lifelong insulin therapy [5]. Although identifying candidates for genetic testing remains difficult, residual pancreatic function and lack of islet autoimmunity are significant clinical markers [17].

Sulfonylureas continue to be the first-line therapy for MODY13 [2, 6, 9]. Here, we report a case of KCNJ11 (p.Arg192Cys)-related MODY13 accompanied by autoimmunity. This finding expands the clinical spectrum of KCNJ11‑related diabetes and highlights the diagnostic and therapeutic challenges in managing the atypical presentations of pediatric diabetes.

## Case presentation

**Fig. 1:**
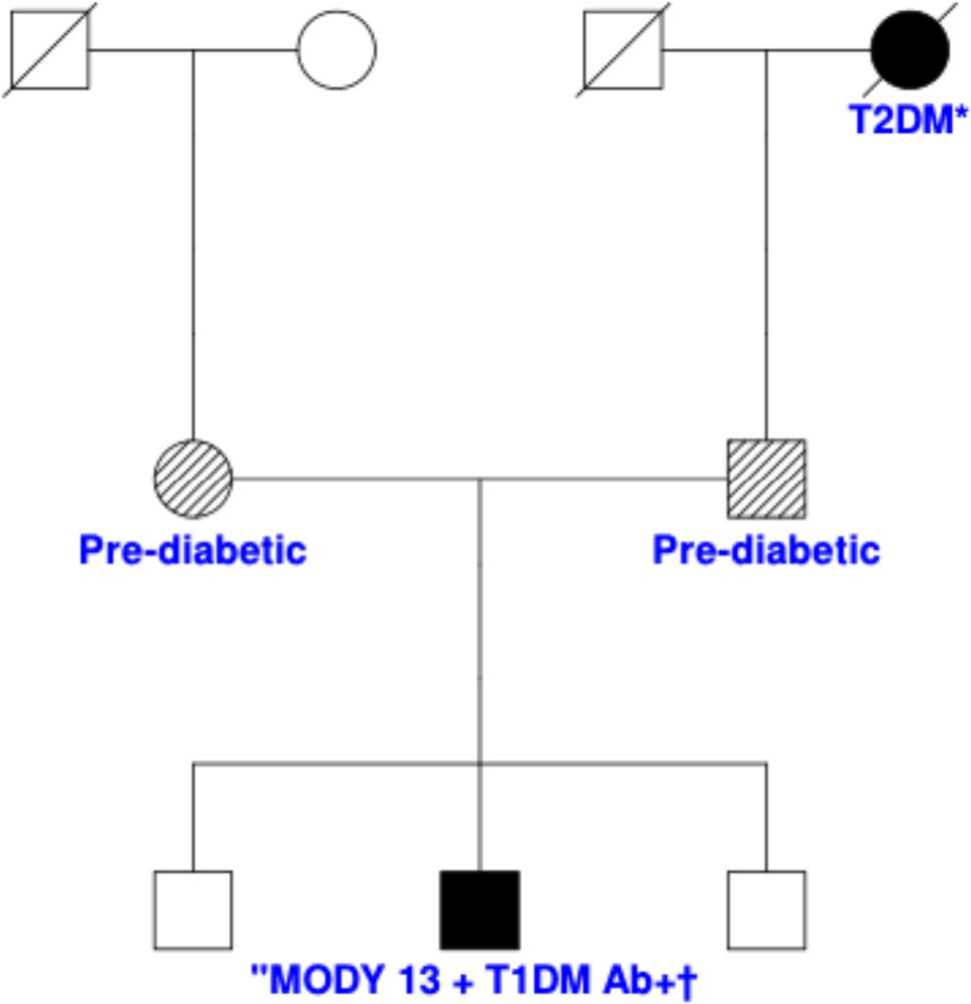
Three-generation pedigree of a patient with MODY13 and coexisting type 1 diabetes autoimmunity. *T2DM: type 2 diabetes mellitus. † T1DM Ab+ = positive type 1 diabetes mellitus autoantibodies

A five-year-old Saudi boy presented with polyuria, polydipsia, progressive weight gain, and acanthosis nigricans. He was born at term to non-consanguineous parents with a normal birth weight. The perinatal course was unremarkable. His developmental milestones aligned appropriately with age. Family history revealed that both parents had prediabetic hemoglobin A1c (HbA1c) levels (6.4% and 5.7%, respectively) and that the paternal grandmother managed T2DM with diet control alone (Fig. 1).

On examination, the child’s blood pressure was 93/56 mmHg, height was 123 cm (99th percentile, + 2.76 standard deviation (SD)), weight was 27.7 kg (> 99.9th, + 2.97 SD), and body mass index (BMI) was 22.5 kg/m² (98th percentile, + 2.03 SD). Acanthosis nigricans was noted on the neck; otherwise, physical examination was unremarkable. Laboratory testing showed a random venous glucose level of 240 mg/dL, HbA1c level of 9.6%, normal venous blood gas, no ketonuria, and a fasting C‑peptide level of 1.14 ng/ml; (normal range: 0.78–5.19 ng/mL). Initial islet autoantibody testing was performed at an outside hospital early in the disease course and prior to referral to our center. The anti-glutamic acid decarboxylase antibody-65 (GADA-65) level was 0.1 IU/mL (normal < 10 IU/mL), while the anti‑insulin antibody (IAA) measured 16.45 UIU/mL (normal up to 10 UIU/mL), and the anti-islet cytoplasmic antibody (ICA) level was greater than 1/5 IU/mL (normal negative). IA-2 A (tyrosine phosphatase antibody) and zinc transporter 8 (ZnT8A) antibody were not assessed.

The patient was managed as a T2DM case with diet and exercise. Over three months, he lost approximately 11 kg, and his HbA1c level decreased to 5.8%. He did not require insulin therapy or develop diabetic ketoacidosis (DKA) episodes during this period. The patient was first evaluated at our institution approximately one year after the initial diagnosis. Given the atypical clinical course with prolonged insulin independence, absence of ketoacidosis, and significant glycemic improvement without pharmacologic therapy, yet still experiencing intermittent postprandial hyperglycemia (> 200 mg/dL) along with stress-induced hyperglycemia, these characteristics raised concerns regarding misclassification of the diabetes type and prompted further evaluation at our institution. Fasting and stimulated C-peptide levels were obtained to assess endogenous insulin reserve, yielding a value of 0.7 ng/ml (reference range: 0.78–5.19 ng/mL), and a stimulated C-peptide level of 1.14 ng/ml (reference range 1.2–6.6 ng/ml). These results were interpreted with the results of the diabetes antibody panel, which yielded seroconversion, including a high GADA‑65 antibody level of 62 IU/mL (normal < 17 IU/mL), an IA-2 A (tyrosine phosphatase antibody) level > 350 IU/mL (normal < 5 IU/mL), ICA positive (normal negative), and zinc transporter 8 antibodies (ZnT8A) > 2000 U/mL (normal < 15 U/mL), confirming autoimmunity. He was subsequently placed on a basal‑bolus insulin regimen (insulin degludec and insulin aspart) at approximately 0.7 U/kg/day; however, his glucose readings remained markedly labile.

Whole-exome sequencing was performed, as it was the only available option for monogenic diabetes testing, and identified a heterozygous autosomal dominant missense variant in KCNJ11 (NM_000525.3:c.574 C > T:p.Arg192Cys), which was classified as likely pathogenic according to the criteria established by the American College of Medical Genetics and Genomics and the Association for Molecular Pathology (ACMG/AMP) (PM2, PP2, PP3) and supported by in silico prediction tools (Varsome and Franklin).

Introducing oral glibenclamide at 0.2 mg/kg/day alongside the insulin regimen lowered his HbA1c level to 7.5% and showed 70% TIR on CGMS. No severe hypoglycemia or adverse effects were observed during the follow-up period. This case illustrates the complex coexistence of MODY13 and autoimmune diabetes.

**Fig. 2:**
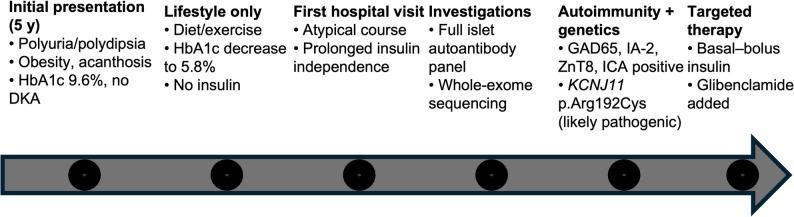
Timeline summarizing the clinical course, diagnostic workup, and treatment milestones in a child with MODY13 and evolving autoimmune diabetes

The sequence of clinical events, diagnostic reassessment, and treatment milestones is summarized in Fig. 2.

### Discussion and conclusions

This case describes a novel KCNJ11 (p.Arg192Cys) variant associated MODY13 with concurrent evolving autoimmune diabetes. The co-occurrence of autoimmunity raises two possibilities: (1) the multifactorial “double diabetes” phenomenon, where genetic susceptibility and immune activation run in parallel, or (2) potential gene–immune interaction wherein adenosine triphosphate-sensitive potassium (KATP) channel dysfunction itself alters the immune trigger. Prior functional studies on other KCNJ11 variants (p.R34H) validated that impaired KATP channel activity induces MODY13 and accounts for the observed sulfonylurea responsiveness [18]. While insulin antibodies can arise following exogenous insulin exposure, this does not explain the subsequent development of other type 1 diabetes-associated autoantibodies, which is consistent with an underlying autoimmune process.

Previous KCNJ11 variants have revealed a wide range of clinical spectra, from hyperinsulinemic hypoglycemia and NDM to MODY13 and T2DM [6, 11, 19–21]. Chen et al. conducted a systematic review of MODY13 cases, reporting a mean age at disease onset of 25.2 years (± 15.3), with the majority of cases manifesting later compared with other MODY subtypes, and displaying a wide-ranging spectrum of metabolic phenotypes [7]. Similarly, He et al. illustrated the diverse clinical presentations linked to KCNJ11-related diabetes, highlighting its clinical heterogeneity and the significance of precise diagnosis for targeted therapy [22]. However, to our knowledge, the coexistence of genetically confirmed MODY13 with persistent type 1 diabetes – associated autoimmunity has not been previously reported. This observation highlights the limitations of excluding monogenic diabetes solely on the basis of islet autoantibody positivity.

Several studies have highlighted the benefits of glibenclamide in KCNJ11-related diabetes. Narasimhegowda et al. reported on a child misdiagnosed with T1DM who achieved satisfactory glycemic control after switching to oral glibenclamide therapy [23]. Similarly, Song et al. [24] and Liu et al. [19] demonstrated significant improvements in glycemic control and the advantages of oral sulfonylureas in KCNJ11-related diabetes. Likewise, additional studies have highlighted the crucial role of genetic diagnosis in fine-tuning the management of MODY13 diabetes [14, 18, 25, 26]. This case corresponds with the literature in that sulfonylurea administration can improve glycemic control; however, this stands out because of the co-presence of marked autoimmunity. This suggests that while glibenclamide may have improved residual β-cell function, total insulin independence was not anticipated given the persistent autoimmune β-cell destruction, highlighting the importance of individualized combination therapy and careful titration.

The limitations of this study include the lack of segregation analysis, absence of parental genetic testing due to financial constraints, lack of local availability, and a relatively short follow-up period. Nonetheless, this case emphasizes the importance of genetic testing for atypical pediatric diabetes and demonstrates how registries can enhance MODY13 recognition in Saudi Arabia. Future studies should explore whether immune activation alters KCNJ11 mutation expression, yielding a coincidentally overlapping picture.

This case demonstrates the diagnostic challenge in pediatric diabetes when the features of T1DM, T2DM, and MODY overlap. Prompt genetic testing can lead to precise therapy. The simultaneous appearance of MODY13 and autoimmunity suggests possible interactions between the gene and the immune system.

## Data Availability

The corresponding author can provide the data used in this study upon reasonable request. The Novel variant described has been submitted to ClinVar and is currently under review.

## Abbreviations

MODY13: Maturity-onset diabetes of the young, type 13
KCNJ11: Potassium inwardly rectifying channel subfamily J member 11
T1DM: Type 1 diabetes
T2DM: Type 2 diabetes
WES: Whole-exome sequencing
NDM: Neonatal diabetes mellitus
HbA1c: Glycated hemoglobin
CGMS: Continuous glucose monitoring system
BMI: Body mass index
GADA: Anti-glutamic acid decarboxylase antibodies
IAA: Anti-insulin antibodies
IA-2A: Anti-islet antigen 2 antibodies
ZnT8A: Anti-zinc transporter 8 antibodies
DKA: Diabetic ketoacidosis
